# An Update on Vitamin D Metabolism

**DOI:** 10.3390/ijms21186573

**Published:** 2020-09-08

**Authors:** Federica Saponaro, Alessandro Saba, Riccardo Zucchi

**Affiliations:** Department of Surgical, Medical and Molecular Pathology and Critical Area, University of Pisa, 56126 Pisa, Italy; alessandro.saba@unipi.it (A.S.); riccardo.zucchi@unipi.it (R.Z.)

**Keywords:** vitamin D, vitamin D receptor, VDR, 1,25(OH)_2_D, 25OH-vitamin D

## Abstract

Vitamin D is a steroid hormone classically involved in the calcium metabolism and bone homeostasis. Recently, new and interesting aspects of vitamin D metabolism has been elucidated, namely the special role of the skin, the metabolic control of liver hydroxylase CYP2R1, the specificity of 1α-hydroxylase in different tissues and cell types and the genomic, non-genomic and epigenomic effects of vitamin D receptor, which will be addressed in the present review. Moreover, in the last decades, several extraskeletal effects which can be attributed to vitamin D have been shown. These beneficial effects will be here summarized, focusing on the immune system and cardiovascular system.

## 1. Introduction

Vitamin D is a steroid hormone which exerts a crucial role in the maintenance of bone and calcium homeostasis. The discovery dates back to one hundred years ago, but vitamin D has become a hot topic in endocrinology research only in the last decades, and it has recently emerged as a burning issue due to the COVID-19 pandemic, because of the alleged correlation between hypovitaminosis D and high risk of chronic pulmonary diseases and mortality [1]. It is now clear that vitamin D displays a complex multistep metabolism and acts as a hormone on many extra-skeletal targets [2]. The aim of this review is to focus on some new, intriguing, and still incompletely clarified aspects of vitamin D metabolism, such as novel concepts in enzyme regulation, new pleiotropic effects of vitamin D receptor (VDR) activation, and epigenetic effects.

## 2. Vitamin D and Skin: from Production to Final Effect

**Figure 1:**
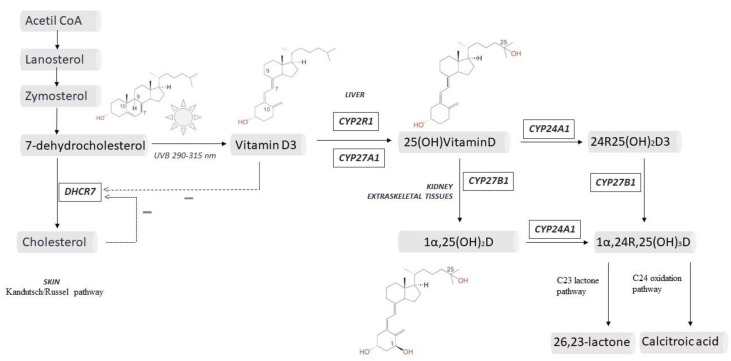
Crucial steps in vitamin D metabolism.

Vitamin D exists in two forms: vitamin D3, which is the most important source in animals and is produced in the skin; and vitamin D2 which differs from D3 for a methyl group in C24 and a double bond in C22–C23 and is produced by plants [3]. In the skin, vitamin D3 is produced from 7-dehydrocholesterol (7DHC), an intermediate in cholesterol synthesis. Exposure to ultraviolet B (UVB) light, in the range of 290–315 nm, determines an electrocyclic rearrangement of the ring in the C9-C10 position, yielding pre-vitamin D (PreD3). Once PreD3 is formed, thermal isomerization to vitamin D3 (VitD3) occurs, with the shift of a hydrogen from C19 to C9 [4] (Figure 1).

This reaction is reversible and PreD3 and VitD3 both coexist. From an evolutionary point of view, the observation that VitD3 production is strictly dependent on UVB sheds light on the ancient origin of the hormone, at least 1.2 billion of years ago, when algae began to produce cholesterol [5]. This process has probably developed as a scavenger mechanism to protect from UVB radiation, which is absorbed and dissipated in the rearrangement of double bonds [6].

As a matter of fact, the synthesis of VitD3 depends on the concentration of 7DHC, which in turn depends on 7 Dehydrocholesterol Reductase (DHCR7) activity. This enzyme catalyzes the reversible reduction of 7DHC into cholesterol. This is part of the biochemical pathway first described by Kandutsch and Russel in 1960 (alternative to the Bloch pathway), in which six isoprene units from acetyl-CoA are converted in a cyclized isoprenoid hydrocarbon (lanosterol) and subsequently through oxidative/reductive steps into zymosterol, zymostenol, 7DHC, and finally cholesterol (Figure 1) [7]. It was only in 2015 that the Kandutsch/Russel pathway was completely elucidated and found to have a high activity in the skin, providing the substrate for VitD3 production [8,9].

In the last fifty years, the discovery of a rare syndrome called Smith-Lemli-Opitz syndrome (SLOS, OMIM #270400) which is caused by mutations in the DHCR7 gene provided interesting information [10,11]. SLOS has an incidence of 1:40,000 and it is clinically characterized by morphogenic and congenital aberrations, with cognitive retardation and altered behavior [12]. Currently 110 different mutations of the DHCR7 gene have been described, that cause enzyme inactivation and accumulation of 7DHC [12,13]. The most frequent mutations are: a null mutation IVS8-1G>C and a nonsense mutation W151X; the others being missense mutations [9]. SLOS is more common in countries with low sun exposure and this observation has been interpreted as an heterozygous advantage for mutation carriers to avoid vitamin D (we will refer to vitD3 if not differently specified) deficiency [14]. Vitamin D levels have been measured in few SLOS patients. In 2005 Rossi et al. did not find any difference in vitamin D levels in 15 patients with SLOS, compared to healthy matched controls [15], but this finding might be attributed to the photosensitivity of SLOS patients, leading to reduced exposure to the sunlight. On the other hand, Movassaghi et al. evaluated 53 pediatric patients with SLOS and found significantly higher levels of 25 hydroxyvitamin D (25OHD), the marker of vitamin D status, across all seasons (48.06 ± 19.53 ng/mL vs. 30.51 ± 16.14 ng/mL, *p* < 0.01), without signs of vitamin D intoxication (normal serum calcium) [16]. Moreover, the genetic locus DHCR7/NADSYN1 was found to be a determinant of vitamin D status in two contemporary Mendelian studies or large-scale genome-wide association studies (GWAS) [17,18], but the hypothesis that some DHCR7 polymorphisms are correlated with vitamin D status is still controversial and has not been confirmed by some studies [9].

Biochemical regulation of DHCR7 seems to be a crucial aspect in vitamin D production, since reduced activity of this enzyme can redirect the pathway from cholesterol to vitamin D biosynthesis. Indeed, at the transcriptional level both vitamin D or cholesterol can reduce DHCR7 expression [19]. At the post-translational level, enzyme phosphorylation seems to be important: Prabhu et al. showed that inhibition of AMP-activated protein kinase and protein kinase A significantly reduced DHCR7 activity, enhancing vitamin D synthesis and reducing cholesterol production [20].

In summary, DHCR7 enzyme is the first line of regulation of vitamin D biosynthesis in the skin, even if the actual production is also modulated by other factors including genetic polymorphisms, age, geographical location and latitude, exposure behavior and cultural conducts, UVB dose, clothing and body surface area (BSA) exposed [1]. Concerning regarding sunscreen photoprotection and vitamin D status have recently demonstrated to be inconsistent by an international panel of experts who reviewed the literature and concluded that vitamin D production is not affected by sunscreen use [21].

The skin has long been known as the major source of vitamin D. Moreover, keratinocytes of epidermis and hair follicles express the hydroxylases needed to produce the active hormone 1,25 dihydroxy vitamin D [1,25(OH)_2_D] (see Section 3), and VDR has been shown to be present on keratinocytes [22]. As a matter of facts, vitamin D produces autocrine and paracrine effects in the skin [23]. In keratinocytes vitamin D has been shown to control differentiation, proliferation, barrier activity and immune response [24]. In selective epidermis VDR knockout animals, predisposition to cancer and impaired wound healing has been observed [25]. Moreover, vitamin D deficiency has been related to skin inflammatory diseases and vitamin D analogues have been found to be effective in psoriasis, a proliferative inflammatory skin disease [26].

## 3. Liver and a New Life for CYP2R1

It is well established that vitamin D requires two subsequent hydroxylation steps to become the active hormone 1,25(OH)_2_ D (1,25-dihydroxy vitamin D, calcitriol). The first hydroxylation, in the C25 position, occurs mainly, but not exclusively, in the liver, with a non-regulated and substrate dependent mechanism, as originally reported. As a matter of fact, several enzymes display 25-hydroxylase activity and among them CYP2R1 has been found to play the major role in the liver and testis. CYP2R1 is located in the microsomal P450 fraction of hepatocytes, as reported quite recently by Cheng et al. [27]. There are few studies on catalytic properties of the enzyme: in a yeast system, Shinkyo et al. demonstrated that CYP2R1 can hydroxylate either VitD3 or VitD2, with higher affinity for the first compared to the latter [28]. Subsequently, this observation has been confirmed in Escherichia Coli and the crystallographic structure of the enzyme has been determined. Notably, the pocket for vitamin D entrance faces the hydrophobic membrane domain [29,30]. The human *CYP2R1* gene is located on chr. 11p15.2 (15.5 kb) and contains 5 highly conserved exons, codifying for a 501-amino acid protein. In *CYP2R1* knockout mice, 25OHD levels have been found to be decreased by about 50%, the other 50% being ensured by other 25-hydroxylase enzymes [31].

In humans, five *CYP2R1* mutations have been identified in patients with different phenotypes, including rickets and low 25OHD levels [32,33,34,35]. Moreover, more than 25 single-nucleotide polymorphisms (SNPs) of *CYP2R1* are known and could explain the population variability in 25OHD concentration observed in some genome-wide association studies [17,18]. Particularly, a recent meta-analysis suggested that the rs10741657 polymorphism has a role in the genetically determined vitamin D deficiency [36].

Despite the previous hypothesis that CYP2R1 is a non-regulated substrate dependent enzyme, recent evidences challenged this dogma, suggesting that the enzyme expression is modulated by age and metabolic environment. 25OHD levels decrease and are less responsive to supplementation in older patients. Roizen et al. attributed this finding to a reduction in CYP2R1 activity in aging, since CYP2R1 mRNA and protein content in hepatic tissue of male mice progressively decreased from 26 to 39 and 49 weeks (one-way ANOVA, *p* = 0.0077). Moreover, the 25OHD3/VitD3 ratio was positively correlated with CYP2R1 mRNA and consistently declined with age [37].

The metabolic layout also affects CYP2R1 expression. It is known that 25OHD levels are significantly reduced in patients with obesity and type 2 diabetes. The current hypothesis is that vitamin D could be sequestered in adipose tissue or diluted in the high surface of obese people. However, a reduction in CYP2R1 activity has been proposed as an alternative explanation. CYP2R1 activity was diminished in an animal model of high fat diet (HFD) obesity, since CYP2R1 mRNA was significantly lower (40%) compared to lean mice, whereas other 25-hydroxylase enzymes were not altered by HFD. CYP2R1 protein expression and enzyme activity was reduced by 50% in obese mice liver homogenates compared to controls [38]. Other investigators observed that 12 h-fasting strongly reduced CYP2R1 mRNA and the effect was even higher after 24 h (50% and 80% respectively), both in mice or rat models [39]. Protein expression and enzymatic activity were reduced accordingly. In different models of diabetes (HFD induced type 2 diabetes and streptozocin induced type 1 diabetes) a similar suppression of liver CYP2R1 activity was observed [39]. At least two potential signaling pathways have been involved in CYP2R1 modulation: the peroxisome proliferator-activated receptor γ coactivator 1-α/estrogen related receptor α (PGC1α/ERRα) and the glucocorticoid receptor (GR) axis. The PGC1α/ERRα pathway is physiologically activated during fasting and it is pathologically induced in diabetes [40,41]: overexpression of this signaling strongly decreased CYP2R1 hydroxylate activity [39]. However, other mechanisms are likely to exist, since suppression of CYP2R1 by starving was observed also in PGC1α knockout mice. Aatsinki et al. showed that pharmacological inhibition of GR prevented CYP2R1 induction by fasting, suggesting a role for this pathway in hydroxylase regulation [39].

These findings suggest a complex crosstalk between vitamin D and several metabolic pathways, so that 25OHD levels undergo a refined control, and do not simply mirror vitamin D intake, as usually assumed. In addition, further hydroxylase activities have been found in the liver. They include CYP27A1, which is located in the mitochondria and has a major role in cholic acids formation [42], and CYP3A4, which has a compensatory C25 hydroxylase activity [43].

## 4. 25(OH)_2_D and the Case for Vitamin D Immunobiology

Besides the well-known role of vitamin D in calcium and bone metabolism, in the last ten years additional effects have been described, with special regard to the immune system. From an evolutionary point of view, specific investigations and genome-wide association studies demonstrated that the ancient and initial role of vitamin D was likely the regulation of genes involved in energy metabolism [5]. During vertebrate evolution, skeletal and immune systems evolved quite simultaneously and vitamin D was a central driver of the osteo-immune bidirectional interactions [44].

As a matter of fact, the primary reason for these extra-skeletal effects of vitamin D is the ability of different tissues to produce the active hormone, i.e., 1,25(OH)_2_D, locally, thanks to the enzyme 1α-hydroxylase. Despite the existence of several 25-hydroxylase enzymes, CYP27B1 has been demonstrated to be the only 1α-hydroxylase in human, and different tissues isoforms exist [45]. It is noteworthy that whereas 25OHD is easily detectable in blood and urine (in the order of ng/mL), the concentrations of 1,25(OH)_2_D are much lower (order of pg/mL) and are largely regulated peripherally with autocrine and paracrine mechanisms, which escape systemic endocrine control and detection.

**Figure 2:**
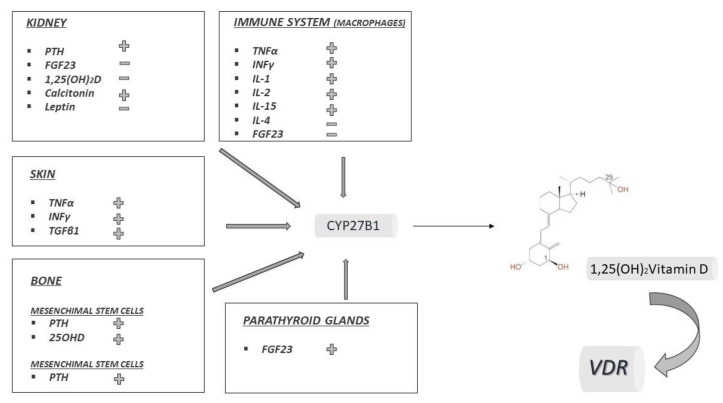
Tissue specific regulation of CYP27B1. In each box factors that stimulate (+) or inhibit (−) CYP27B1 are represented.

In 1971, renal CYP27B1 was identified and the kidney was thought to be the only source of 1,25(OH)_2_D [46]. The renal form of CYP27B1 is regulated by at least three hormones, with a crucial role in calcium-bone metabolism (Figure 2): parathyroid hormone stimulates the hydroxylation, whereas FGF23 and 1,25(OH)_2_D itself inhibit it, in response to calcium and phosphate concentrations [47,48]. Calcitonin has also been shown to stimulate renal CYP27B1 and leptin to inhibit, probably via FGF23 (Figure 2) [49].

Beyond classical renal CYP27B1 modulation, the novelty in the field is represented by a completely different regulation of CYP27B1 in the other tissues, particularly in the immune system. In the 1980′s, it was observed that the administration of 1,25(OH)_2_D to blood myeloid cells induced their maturation into white cells [50]. The contemporary report of hypercalcemia and high levels of 1,25(OH)_2_D in an anephric patient with sarcoidosis, suggested that C1 hydroxylation could occur outside the kidney [51]. In 1983, Adams et al. observed 1,25(OH)_2_D production from macrophages in sarcoidosis patients [52]. It is now known that macrophages are involved in the pathophysiology of many inflammatory and/or autoimmune diseases (sarcoidosis, tuberculosis, Chron’s disease, foreign body granulomata, cryptococcosis and others), and that they are able to produce 1,25(OH)_2_D at high levels by their own CYP27B1 [53]. Differently from renal CYP27B1, the macrophage isoform is not controlled by PTH. 1,25(OH)_2_D formation depends only on substrate availability and is not limited by product accumulation, which was interpreted as absence of catabolic enzymes control [52,53]. This is likely the reason why in sarcoidosis 1,25(OH)_2_D production is persistent and eventually leads to systemic hypercalcemia [54]. The regulation of CYP27B1 in macrophages and monocyte has been elucidated and it is under the control of cytokines and inflammation. Macrophages’ CYP27B1 is stimulated by interferon-γ (INFγ), tumor necrosis factor α (TNFα), interleukin (IL) 1, 2 and 15, but not by PTH. Moreover, dexamethasone inhibits CYP27B1 [55]. In addition to macrophages, also dendritic cells (DC), Th lymphocytes and B lymphocytes express CYP27B1, but only when they are activated. In these cells 1,25(OH)_2_D functions as a 1αhydroxylase inhibitor, thus controlling their activation and proliferation. As described further in details in paragraph 6, 1,25(OH)_2_D exerts many autocrine and paracrine functions on immune system cells, ensuring a feedback control on immune cells themselves [55].

Local production of 1,25(OH)_2_D by CYP27B1 for autocrine/paracrine purpose has been described in many other tissues, including epithelial tissues, placenta, bone, endocrine glands (parathyroid, pancreatic islets, thyroid, adrenal medulla, gonads), brain, liver and endothelia [53]. In the majority of cases, experimental data suggest that the regulation of local CYP27B1 escapes the classical patters of the renal isoform and is due to local tissue-specific stimuli [53]. In Figure 2 the main regulators of extrarenal CYP27B1 isoforms are summarized.

## 5. Vitamin D Catabolism, Metabolites and Transport

More than 50 metabolites of vitamin D have been described in the last decades and some of them display a certain interest because of their biological activity. The best-known catabolic enzyme is CYP24A1, belonging to mitochondrial P450 fraction and encoded by the *CYP24A1* gene on chr. 20q13.2 [48]. CYP24A1 can hydroxylase both 25OHD and 1,25(OH)_2_D, producing 24R,25(OH)_2_D and 1,24,25(OH)_3_D, respectively. The same enzyme further catalyzes the hydroxylation of these products in multiple steps, yielding a series of 24—and 23—hydroxylated derivatives. The final products are the inactive calcitroic acid or 26,23-lactone excreted with bile or urine (Figure 1). CYP24A1 is up-regulated by calcitriol and FGF23 and is inhibited by PTH and hypocalcemia. CYP24A1 has been detected in many tissues expressing VDR, and it plays a crucial role in the local modulation of vitamin D activity [56]. Pathogenic variants of CYP24A1 have been described and they are responsible for Idiopathic Infantile Hypercalcemia (IIH, OMIM 143880), a rare disorder due to impaired vitamin D catabolism and subsequent hypercalcemia. In particular, biallelic variants (in homozygosis or heterozygosis) have a severe phenotype with hypercalcemia that may occasionally lead to death in infant age [57].

In addition to CYP24A1, other minor metabolic pathways have been described and still need further evaluation. Recently, the C-3 epimerization pathway has been identified, which leads to the production of several C-3 epimer metabolites, in which the hydroxyl group on C3 has the alpha rather than the beta orientation in the space. Epimeric metabolites have been shown to be highly expressed particularly in neonates and young children, but the physiological role of this redundant pathway needs to be elucidated [58].

The presence of so many metabolites is stimulating the development of novel and more accurate analytical techniques. Since 1970, when high performance liquid chromatography techniques have been introduced, they have been continuously improved and today the most recent LC-MS-MS assay is referred as the “gold standard” method [59,60,61,62]. This is due to the high sensitivity, reproducibility and accuracy, this latter also being influenced by the capability to discriminate 25OHD2 and 25OHD3, as well their epimeric forms. Moreover, it offers the possibility to measure different vitamin D metabolites at the same moment [63]. Right now, five intermediates have been measured with standardized techniques, namely: vitamin D, 25OHD, 1α,25(OH)2D, 24R,25(OH)2D and C3-epi25(OH)D and procedures are listed in the Joint Committee for Traceability in Laboratory Medicine (JCTLM) database [64,65,66,67].

In 2009 the use of LC-MS-MS for vitamin D metabolites measurement was advised by the Nutritional Health and Nutrition Examination Survey [68] in USA and from the UK Food Standard Agency (FSA) in their National Diet and Nutrition Survey [69].

The hope is that with LC-MS-MS further insight in the complexity of vitamin D metabolites will be achieved [70,71].

Transport of vitamin D metabolites is accounted for 85% by vitamin D binding protein (DBP) with high affinity and 15% by albumin with low affinity [72].

DBP, initially known as Gc-globulin, is a multitasking protein which is very conserved in vertebrates’ evolution. The human gene for DBP is located on chr. 4, close to other genes for albumin family proteins and encodes for a 458 amino acid single chain protein [73]. All metabolites of vitamin D can be bound by the same binding site of DBP, even if 25OHD and 1,25(OH)_2_D have the highest affinity [74].

Free 25OHD represents 0.03% and 1,25(OH)_2_D 0.4% of the total amount of the metabolites and have been classically interpreted as the only active hormone to enter cells (free hormone hypothesis [75]). However, at least in some organs like kidney, the free hormone hypothesis has been recently revised. Indeed, it has been shown that the large transmembrane protein megalin is present on the apical side of the proximal tubule cells and acts as a receptor for the complex vitamin D-DBP, together with cubulin and disabled-2 proteins [76]. Accordingly to this hypothesis, knockout mice for Lrp2 (encoding for megalin) show severe osteomalacia and poor survival, demonstrating the pivotal role of DBP binding capacity in the kidney [77]. On the other hand, the role of this mechanism in the other tissues is still debated: megalin is expressed in several tissues in which vitamin D exerts extraskeletal functions, but Megalin-mediated uptake of DBP has not been completely elucidated. Summarizing recent evidences, DBP functions as a large pool reservoir of circulating 25OHD, which prevents for vitamin D deficiency when supply is low. Moreover, DBP also functions as a regulator for vitamin D access to cells in kidney and most likely in the other peripheral tissues [72].

## 6. VDR: The History of a Nutrient that Controls Several Genes and the Epigenetic Modulation

The human vitamin D receptor gene (VDR) is located on chr. 12 and contains nine exons. In the last twenty years VDR cDNAs were obtained and cloned from several species (human, mice, rats, chicken, frog, quail), revealing a great homology among species and many conserved regions [78,79]. VDR is a polypeptide of 50,000 Da formed by a single amino acid chain. It is almost ubiquitous in the body since it is expressed in at least thirty tissues, involved in bone metabolism (intestine, bone, cartilage, kidney) or in other extra-skeletal functions (heart, immune system, adipose tissues and many others) [80,81]. VDR belongs to the nuclear receptor superfamily along with the receptors of other steroid hormones. These receptors share the ability to bind their ligands at nanomolar concentrations in a specific conserved ligand binding domain (LBD), with a pocket of 400–1400 A3 [82,83]. When VDR binds to 1,25(OH)_2_D, it can reach the nucleus and forms a heterodimer with retinoid X receptor (RXR), able to interact with gene response elements. This interaction is crucial for assembling the transcriptional machinery at the promoters of 1,25(OH)_2_D targets genes [84]. The crystallographic structure of the receptor bound to its major ligand 1,25(OH)_2_D was resolved by Rochel et al. [85]. The ligand binding domain (LBD) is formed by 12 α- helices (H1-12) packed in three layered α helical sandwich and three stranded β sheets. When the ligand binds, H12 is able to shift and deeply closes the ligand into the pocket binding site [85]. The DNA binding domain is formed by two zinc fingers, where four cysteines residues maintain zinc in a tetrahedral configuration [78].

More than 3% of the entire genome from zebrafish to human is under direct or indirect VDR control, so that more than 11,000 genes have been identified as putative targets for VDR, controlling many pivotal mechanisms such as metabolism, cells adhesion, tissue differentiation, development and angiogenesis [86]. Some of the major signaling pathways activated by VDR are summarized in Table 1.

As a matter of fact, vitamin D has recently become a hot topic in nutrigenomics that is the discipline studying the environmental factors able to affect the transcriptome and the epigenome.

The latter is a novel and interesting field in vitamin D research. Chromatin is the structure in which genomic DNA, nucleosome-forming histone proteins and non-histone proteins are packed in the nucleus, and it represents the scaffold of the entire human heritable information [79]. Chromatin exists in at least two different forms: less dense and transcription-available euchromatin and compact, functionally repressed heterochromatin. These different conformations are largely related to post-translational changes of chromatin proteins. Epigenomic studies all the modifications (such as histone methylation or acetylation) which occur in chromatin in the absence of genomic changes [87]. These epigenetic alterations can be very stable and heritable or unstable and transient and are catalyzed by the so-called chromatin modifier enzymes. A few hundred genes for chromatin modifier enzymes have been described, which as Carlberg brilliantly wrote: “add (write), interpret (read) or remove (erase) post translational histone modifications” [88]. VDR acts as a transcription factor and is able to modulate genes encoding for chromatin modifier enzymes, thus modulating the human epigenome. One example is KDM6B/JMJD3, a histone H3 lysine demethylase, which plays a crucial role in development. It has been shown to be induced by 1,25(OH)_2_D/VDR and in turn to modulate vitamin D metabolism. Pereira et al. showed that 1,25(OH)_2_D/VDR induced JMJD3 RNA in human colon cancer cells, suggesting a role for 1,25(OH)_2_D in colon cancer epigenomic events [89].

Another level of epigenomic control by VDR is the direct interaction between VDR and chromatin proteins. Furthermore, VDR has been shown to interact with co-activators, such as those of the NCOA family, or co-repressors, such as NCOR1 proteins, which respectively lead to local chromatin opening or closing [90]. The enrolment of co-regulators is also important in the tissue specific and cell-specific regulation of differentiation. An example is the skin were the change of different 1,25(OH)2D/VDR genes controlling the process of differentiation of the keratinocytes is regulated by the subsequent recruitment of different co-regulators of the mediator complex family (MED) in the early stage and steroid receptor co-activator (SRC3) in the later stages [23].

VDR seems to interact with more than 50 nuclear proteins, and deeply affects chromatin remodeling [91].

VDR has also been detected in a different subcellular location, namely as a transmembrane receptor which appears to be activated by vitamin D analogues with a different configuration (6S-cis), if compared to those that activate the nuclear VDR (6S-trans). Transmembrane VDR is one of the receptors by which vitamin D exerts non genomic, rapid effects on its target cells and tissues. Some non-genomic effects of vitamin D, which occur rapidly over minutes or hours, include rapid intestinal absorption of calcium (transcaltachia), secretion of insulin by pancreatic cells, opening of voltage-gated Ca2+ and Cl- channels in osteoblasts, and the rapid migration of endothelial cells [92].

## 7. Pleiotropic Effects of Vitamin D

**Figure 3:**
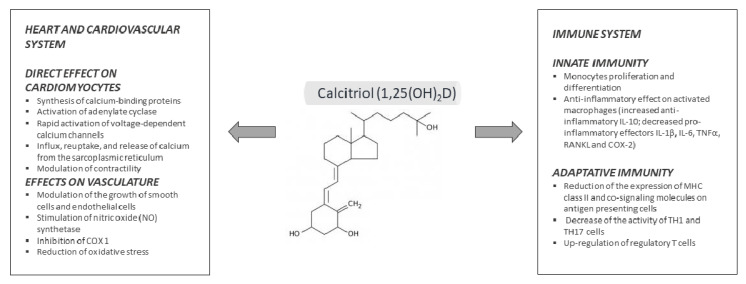
Pleiotropic effects of calcitriol on immune system and cardiovascular system.

The classical role of 1,25(OH)_2_D in calcium/bone metabolism, namely the regulation of intestinal calcium absorption, renal calcium reabsorption and mobilization of calcium and phosphate from bone, has been known for decades and is beyond the aims of the present review. On the other hand, in the last decade a mean of 3000 papers per year have been published on vitamin D, pushed by new findings concerning extra-skeletal effects of this hormone [93]. In this section we will summarize part of these discoveries and the effects of 1,25(OH)_2_D on several districts, particularly the immune system and the cardiovascular system (Figure 3). Actions of vitamin D on skin have been summarized in the first paragraph.

*Immune system*: Vitamin D seems to influence both the innate and the acquired immune system with complex effects, which are still not completely elucidated. We have already outlined that 1 α-hydroxylation produces the active hormone within different cells of immune system, where it exerts autocrine and paracrine effects. A very recent study on vitamin D target genes evaluated and compared all the available transcriptome-wide datasets from human monocytes treated with 1,25(OH)_2_D in vitro [94]. 15 VDR target genes with a potential pivotal role in the immune response were identified and classified in three groups. Group 1 included *CAMP, CD14, FN1*, and *TREM1* genes, which have a low basal expression but are highly inducible after 1,25(OH)2D/VDR activation and encode proteins involved in the immediate response to infection. In general, effectors of the LPS/TLR4 signaling pathways seem to be coded by these genes. Group 2 includes *LILRB4, LRRC25, MAPK13, SEMA6B, THBD*, and *THEMIS2* genes, which are required in the general response to infection. Group 3 includes *ACVRL1, CD93, CEBPB, NINJ1, SRGN*, genes involved in long-term autoimmunity mechanisms, which do not require ligand binding to VDR and are likely involved in epigenomic regulation [94].

Mathieu et al. recently reported a comprehensive revision of vitamin D effects on the immune system [95]. Both monocytes and macrophages express VDR, the latter at higher levels than the former. 1,25(OH)_2_D has been shown to stimulate differentiation and proliferation of monocytes, whereas on activated macrophages the overall effect leads to reduced inflammatory response. Indeed, 1,25(OH)_2_D stimulates the production of IL-10 (anti-inflammatory) and decreases the release of pro-inflammatory effectors such as IL-1β, IL-6, tumor necrosis factor-α (TNFα), receptor activator of nuclear factor kappa-Β ligand (RANKL), and cyclo-oxygenase-2 (COX-2) [96]. The signaling pathways proposed to mediate the anti-inflammatory effect include: (i) upregulation of MAPK and MKP and inhibition of LPS/p38, (ii) modulation of thioesterase superfamily member 4, with subsequent COX2 inhibition, (iii) direct antimicrobial effect by cathelicidin antimicrobial peptide (CAMP) induction, (iv) anti-oxidative effect due to increased glutathione reductase (GR) with drop of reactive oxygen species [95,96].

On the other hand, vitamin D inhibits the acquired immune system, mainly reducing the expression of MHC class II and co-signaling molecules on antigen presenting cells, decreasing the activity of TH1 and TH17 cells, and up-regulating regulatory T cells. The final result is to promote the regulatory and protective phenotype of T cells [97].

From a clinical perspective, vitamin D deficiency is strongly associated with increased risk of infections, dysregulation of the immune system and autoimmune diseases [1].

A specific issue that recently gained a great relevance is the relationship between hypovitaminosis D and pulmonary infections. Vitamin D concentration is inversely related to the risk of multiple pulmonary injuries such as pneumonia, community acquired pneumonia, ARDS, sepsis, heart failure and mortality from pulmonary infections [98,99,100,101,102]. A recent large metanalysis on more than 10,000 subjects demonstrated that vitamin D supplementation had a protective role in acute respiratory infections, in adults [103]. Due to these findings, an important role for vitamin D has very recently been suggested in the treatment or prevention of COVID-19. In March 2020, COVID-19 spread as a pandemic emergence due to the new *β* Coronavirus Severe Acute Respiratory Syndrome Coronavirus 2 (SARS-CoV-2) [104]. In the absence of specific treatments, public health measures are required to characterize risk factors and prevent the infection or the progression of the disease. Among the factors that might contribute to the development of severe COVID-19, vitamin D status was proposed as a credible candidate [105,106], even if the evidence is still preliminary. At present, the following observations suggest a possible role of vitamin D in reducing SARS-CoV-2 risk: (i) the seasonal flare of COVID-19, which coincides with the nadir of vitamin D levels, (ii) the previously mentioned association between hypovitaminosis D and pulmonary infections, (iii) the anti-inflammatory role of vitamin D which could be of benefit against the so called “cytokine storm”, which seems to be a pathophysiological pivotal player in SARS-CoV2 morbidity and mortality [107]. Several short reports have been published and are still emerging at the moment of this writing, encouraging to analyze the relationship between vitamin D and COVID-19 [105,106,108].

In the setting of autoimmune diseases, there is an interesting association between low levels of vitamin D and increased risk of developing multiple sclerosis (MS). Some prospective studies have demonstrated that increasing levels of 25OHD significantly reduce the risk of MS, among Caucasian people [109]; moreover, cholecalciferol supplementation associated with interferon β1b significantly reduced the activity of the disease as evaluated by MRI, compared to interferon β1b alone [110].

*Cardiovascular system:* In the early 1980s, Robert Scragg proposed the hypothesis that the increase in cardiovascular diseases usually observed in winter might be a result of low 25OHD levels, due to the reduced sunlight exposure [111]. This idea turned on a great interest in the potential cardiovascular benefits of vitamin D, leading to several publications over the last ten years. However, the physiological role of vitamin D in the cardiovascular system is still unclear.

VDR is expressed in rat and human heart tissue and has a potential role as a modulator of cardiac hypertrophy and failure. This hypothesis is based on the concept that altered intracellular handling of ionized calcium is related to the impaired contractility of the myocardium in heart failure (HF), since 1,25(OH)_2_D is directly involved in calcium-dependent cellular processes, including synthesis of calcium-binding protein, activation of adenylate cyclase, rapid activation of voltage-dependent calcium channels, and modulation of sarcoplasmic reticulum calcium uptake and release [112].

Another possible mechanism is the putative role of 1,25(OH)_2_D as a negative regulator of the renin-angiotensin system (RAS). In both normotensive and hypertensive subjects, 1,25(OH)_2_D serum levels are inversely associated with PRA (plasma renin activity), suggesting a potential role of vitamin D in hypertension via renin regulation. Recent evidence showed that nuclear hormone receptors, including VDR, liver X receptor (LXR) and peroxisome proliferators-activated receptor (PPAR), regulate renin gene transcription via specific elements in the renin promoter [112].

Strong support for the involvement of vitamin D in the pathogenesis of cardiovascular diseases comes from VDR knockout mice (VDR^−/−^). These mice develop typical signs of HF, including activation of the renin–angiotensin–aldosterone system, cardiac hypertrophy, high blood pressure, and increased levels of atrial natriuretic peptide. Furthermore, the development of hypertension in VDR^−/−^ mice can be corrected with the administration of ACE inhibitors, only as long as vitamin D levels are sufficient [113,114,115].

Additional effects of 1,25(OH)_2_D concern the vasculature; indeed, vitamin D can modulate the growth of smooth muscle and endothelial cells and can induce the activation of vasodilatory and antithrombotic genes [116]. Moreover, vitamin D seems to suppress inflammation and to reduce update of oxidized LDL, giving potential vascular benefits. VDR^−/−^ mice show hypercoagulability and atherosclerosis [117,118].

From a clinical point a view, low serum 25OHD levels have been associated with increased risk of cardiovascular diseases, including hypertension, coronary artery disease, ischemic heart disease, HF, stroke, and type 2 diabetes [119,120,121,122,123,124]. Convincing data has been produced particularly on the association between hypovitaminosis D and HF, which still is a major public health disease with poor prognosis. A large cohort of patients with HF has been evaluated by Gotsman et al. who described the variations of serum 25OHD during the year and the effect of vitamin D deficiency and supplementation on mortality. In their population, vitamin D deficiency strongly predicted low survival rate and vitamin D supplementation succeeded in reducing mortality [125]. Our group recently reported results from an Italian cohort of 261 patients with HF in whom low serum levels of 25OHD were inversely correlated with a validated score of HF mortality, namely the Metabolic Exercise Cardiac Kidney Index or MECKI score [126]. In a subgroup of these patients, cardiovascular outcomes have been also reported: patients with HF had mean serum levels of 25OHD statistically lower than healthy subjects (45.2 ± 23.7 nmol/L vs. 58.2 ± 24.0 nmol/L, *p* < 0.001) and a higher prevalence of vitamin D insufficiency (serum 25OHD < 50 nmol/L or 20 ng/mL) (61.1% vs. 39.5%, *p* < 0.001), associated with higher mortality risk [127].

Several meta-analyses on retrospective studies [119,128,129,130,131,132,133] confirmed a consistent association between a low vitamin D status and cardiovascular endpoints (myocardial infarction, hypertension, HF) and/or mortality. On the other hand, randomized controlled trials (RCTs) and Mendelian randomization studies so far have not succeeded in proving a benefit of vitamin D supplementation. However, it is likely that the latter investigations are affected by some methodological limitations (no clear and homogeneous dosage of vitamin D, no measurement of basal 25OHD levels, design of the study targeted on skeletal outcome) therefore it is still unclear if vitamin D has a causative role in cardiovascular diseases or is rather a marker of poor health in chronic disease.

*Adipose tissue and glucose/lipid metabolism*: recent studies showed an association between low levels of vitamin D and almost all aspects of the metabolic syndrome, namely type 2 diabetes mellitus (T2DM), impaired fasting glucose, hypertension, dyslipidemia, obesity, and insulin resistance. Therefore, several investigations focused on the role of vitamin D in adipose tissue biology. Some studies have shown a negative correlation between vitamin D and leptin or resistin, as well as and an inverse correlation with adiponectin. [134,135] Old preclinical studies have demonstrated that a normal activity of pancreatic β cells required adequate levels of vitamin D and functioning VDR [136], while more recent studies on vitamin D deficient mice showed impaired glucose-stimulated insulin secretion in pancreatic islets [137].

A recent review by Mathieu supported role of vitamin D in diabetes. Indeed, VDR is present in all tissues involved in T1 and T2DM, either in pancreatic islets and immune cells, adipose tissue, liver and muscle. In all these tissues and organs, the machinery responsible for the local production of 1,25(OH)2D and its metabolites is also present. Moreover, low levels of vitamin D are associated with increased risk of T1 and T2DM and in several animal models vitamin D supplementation improved islets function and insulin sensitivity. However, convincing randomized prospective studies in humans are still lacking and necessary.

*Muscle:* it has been ascertained that vitamin D deficiency is responsible for low muscle strength, balance disorders and an increased risk of falls. In a model of VDR null mice, a specific phenotype due to immature muscle-specific genes developed and cardiomyocyte-specific VDR knockout induced cardiac hypertrophy and failure [138,139]. Observational studies have confirmed as association between low vitamin D levels and muscle weakness in children and in the elderly, while supplementation improved muscle function as well as energy recovery after exercise [140,141].

*Cancer:* VDR has been shown to be expressed by cancer cell lines and it is hypothesized to play a role in the pathogenesis and progression of cancer. CYP27B1 is also expressed at high level in many cancer cells and tissues and 1,25(OH)_2_D has been proved to have an anti-proliferative effect firstly on myeloma and melanoma cells and subsequently on other cancerous cell lines [142]. On the other hand, CYP24A1 is overexpressed in cancer and several vitamin D analogues and/or CYP24A1 inhibitors have been tested in preclinical studies [143]. Animal models with VDR knockout do not spontaneously develop neoplasia, however, they have been demonstrated to be more susceptible to develop malignancies under stimulus [144] and in several studies on different animal models vitamin D analogues slowed cancer progression and the development of metastases [145]. An interesting example is that of melanomas: 1,25(OH)_2_D and analogues have been clearly showed a beneficial effect as inhibitors of proliferation, plating efficiency and anchorage-independent growth of melanomas cells in vitro experiments and in vivo animal models and have been proposed as potential adjuvant therapies, especially if topically delivered [146,147].

Clinical data regarding vitamin D and cancer are more controversial: low vitamin D levels have been associated with a higher risk of cancer, but RCTs have not been revealed any significant benefit from vitamin D supplementation. However, international multicenter RCTs are still ongoing, specifically designed for the extra-skeletal effects of vitamin D, and they will hopefully shed light on the controversies [1].

## 8. Conclusions and Remarks

Vitamin D has been clearly recognized to be a molecule with several endocrine, paracrine and autocrine effects on multiple tissues and organs, beyond skeletal homeostasis maintenance. Research is still very active in this field, with the aim of clarifying many aspects of the complexity of vitamin D and its metabolites. New concepts have emerged in the last years, namely the special role of the skin, the metabolic control of liver hydroxylase CYP2R1, the specificity of 1α-hydroxylase in different tissues and cell types and the genomic, non-genomic and epigenomic effects of VDR. Many issues need further investigation and many questions are still waiting for answers, which will hopefully become available in the near future.

## Figures and Tables

**Table 1:** Major signalling pathways activated by VDR.

Significant Vitamin D Target Components of Intracellular Signalling
▪ Cell proliferation Cyclin D, p21, p27, GADD45 ▪ Cell signalling AMPK, Beclin-1, CASR, Cathelicidin, DDIT4, PTEN, DICKKOPF-1 ▪ Antioxidant effect G6PD, Gpx, TR ▪ Calcium signalling Calbindin, Ca v1.2, NCX1, PMCA, TRPV5, TRPV6 ▪ Epigenetic components JMJD1A, JMJD3, LSD1, LSD2
